# ^18^F-fluoride Positron Emission Tomography Measurements of Regional Bone Formation in Hemodialysis Patients with Suspected Adynamic Bone Disease

**DOI:** 10.1007/s00223-013-9778-7

**Published:** 2013-08-31

**Authors:** Michelle L. Frost, Juliet E. Compston, David Goldsmith, Amelia E. Moore, Glen M. Blake, Musib Siddique, Linda Skingle, Ignac Fogelman

**Affiliations:** 1 Osteoporosis Unit, Division of Imaging Sciences and Biomedical Engineering, King’s College London, Guy’s Hospital Campus, Great Maze Pond, London, SE1 9RT UK; 2 Department of Medicine, Addenbrooke’s Hospital, University of Cambridge, Hills Road, Cambridge, UK; 3 Renal Medicine, Guy’s & St Thomas’ NHS Foundation Trust, Guy’s Hospital Campus, Great Maze Pond, London, UK

**Keywords:** Adynamic bone disease, ^18^F-fluoride PET, Chronic kidney disease, Osteoporosis, Bone histomorphometry, Bone formation

## Abstract

^18^F-fluoride positron emission tomography (^18^F-PET) allows the assessment of regional bone formation and could have a role in the diagnosis of adynamic bone disease (ABD) in patients with chronic kidney disease (CKD). The purpose of this study was to examine bone formation at multiple sites of the skeleton in hemodialysis patients (CKD5D) and assess the correlation with bone biopsy. Seven CKD5D patients with suspected ABD and 12 osteoporotic postmenopausal women underwent an ^18^F-PET scan, and bone plasma clearance, *K*_i_, was measured at ten skeletal regions of interest (ROI). Fifteen subjects had a transiliac bone biopsy following double tetracycline labeling. Two CKD5D patients had ABD confirmed by biopsy. There was significant heterogeneity in *K*_i_ between skeletal sites, ranging from 0.008 at the forearm to 0.028 mL/min/mL at the spine in the CKD5D group. There were no significant differences in *K*_i_ between the two study groups or between the two subjects with ABD and the other CKD5D subjects at any skeletal ROI. Five biopsies from the CKD5D patients had single tetracycline labels only, including the two with ABD. Using an imputed value of 0.3 μm/day for mineral apposition rate (MAR) for biopsies with single labels, no significant correlations were observed between lumbar spine *K*_i_ corrected for BMAD (*K*_i/BMAD_) and bone formation rate (BFR/BS), or MAR. When biopsies with single labels were excluded, a significant correlation was observed between *K*_i/BMAD_ and MAR (*r* = 0.81, *p* = 0.008) but not BFR/BS. Further studies are required to establish the sensitivity of ^18^F-PET as a diagnostic tool for identifying CKD patients with ABD.

## Introduction

Approximately 6 % of the population of Europe and the United States have moderate to severe renal impairment, and the incidence of end-stage renal disease is now as high as 200 cases per million in many countries [1]. As well as the biochemical alterations and increased vascular calcification that occur in chronic kidney disease (CKD), bone abnormalities are common, starting in those with CKD2 and found in nearly all patients with CKD5 [2, 3]. Fracture risk in patients with a glomerular filtration rate (GFR) of <60 mL/min/1.73 m^2^ is double that observed in those with normal renal function [4]. Reduced bone mineral density (BMD) itself has been associated with an increased risk of all-cause mortality in patients on hemodialysis [5]. At one end of the osteodystrophy spectrum is adynamic bone disease (ABD), which is characterized histologically by low rates of bone turnover, reduced osteoid seam width, and diminished cellular activity [6, 7]. Diagnosing ABD is important since its prevalence is increasing [3], and it is associated with skeletal pain [6], hypercalcemia [8], vascular calcification [9], increased fracture risk [10, 11], and excess morbidity and mortality [12, 13].

As emphasized in the recent Kidney Disease: Improving Global Outcomes (KDIGO) guidelines, bone biopsy remains the gold standard for the diagnosis of the subtypes of renal osteodystrophy [14–16]. However, it is subject also to important limitations including being invasive, being limited to one skeletal site, and requiring considerable expertise at both the time of tissue collection and subsequent quantitative histomorphometry and interpretation [17, 18]. In clinical practice nephrologists therefore have come to rely on serum measurements of intact parathyroid hormone (iPTH) as a surrogate marker of underlying bone disease. Although measurements of iPTH allow reasonable discrimination of ABD from high bone turnover, their ability to correctly classify histology-derived bone formation rates in an individual is extremely limited [19, 20]. Other biomarkers, either alone or in combination with iPTH, have been evaluated; but none has yet proved superior to PTH for the noninvasive prediction of bone histology [19, 21–24].

The functional imaging technique of ^18^F-fluoride positron emission tomography (^18^F-PET) [25] allows the noninvasive assessment of regional bone formation [26, 27] and overcomes the important limitations of conventional techniques: it allows an assessment of regional bone formation at clinically relevant sites and, unlike bone biopsy, is noninvasive and can be readily applied in a clinical setting. Quantitative PET imaging and measurement of the arterial plasma input function allow the bone plasma clearance (*K*_i_) to be calculated. It has been shown that *K*_i_ correlates closely with histomorphometric parameters, including bone formation rate (BFR) and mineral apposition rate (MAR), and therefore provides a quantitative assessment of regional bone formation [26, 27]. ^18^F-PET has been used to investigate regional bone formation in patients with metabolic bone disease, including those with osteoporosis, Paget disease, and end-stage renal disease [28–33]. A number of studies have also demonstrated that it is possible to quantify the direct effects of pharmacological treatments for osteoporosis and other metabolic bone diseases on bone formation at the spine and hip [31, 34–37].

To date, there has been only one study using ^18^F-PET to evaluate bone formation in patients with CKD [26]. This study demonstrated that ^18^F-PET was able to discriminate between those with low-turnover disease and those with secondary hyperparathyroidism [26]. The aims of the current study were to examine regional bone formation at multiple skeletal sites in CKD5D patients with suspected ABD and to assess the correlation between bone formation estimated using ^18^F-PET and histomorphometric indices of bone formation.

## Methods

### Patients

The study population consisted of 19 subjects including seven patients with CKD5 on hemodialysis with suspected ABD (CKD5D group) and 12 healthy ambulatory postmenopausal women with osteoporosis (osteoporosis group). Inclusion criteria for those in the CKD5D group were postmenopausal women aged over 45 years or men aged over 35 years with CKD5 (estimated GFR <15 mL/min/1.73 m^2^), on chronic maintenance dialysis for at least 6 months, and suspected ABD based on iPTH levels <150 pg/mL and calcium levels within the normal range on at least two occasions during the 3 months prior to screening. Patients could continue taking any phosphate binders and vitamin D or its active metabolites prescribed by their treating nephrologist. Exclusion criteria for those in the CKD5D group included diseases known to influence bone metabolism (other than CKD metabolic bone disease), active or chronic liver disease, malignancy, thyroid disease, current use of calcimimetics or anticoagulation therapy, and current use of drugs known to affect bone metabolism (including glucocorticoids, hormone replacement therapy, selective estrogen receptor modulators, or anticonvulsants), and current or previous use within 2 years of screening of bisphosphonates. Inclusion criteria for those in the osteoporosis group included women aged over 45 years, at least 5 years postmenopausal, with osteoporosis defined as a *T* score of ≤2.5 SD below the young adult mean at the lumbar spine, femoral neck, and/or total hip [38]. Exclusion criteria included diseases known to influence bone metabolism (other than osteoporosis), current anticoagulation therapy, current or previous use of bisphosphonates within 2 years of screening, and current use of drugs known to influence bone metabolism (including glucocorticoids, hormone replacement therapy, selective estrogen receptor modulators, or anticonvulsants). All patients had four study visits over a mean duration of 12 weeks: a screening visit to assess suitability for study inclusion including routine laboratory assessments, vital signs, medical history, physical examination, and dual-energy X-ray absorptiometric (DXA) scan of BMD (visit 1); an assessment of biochemical markers of bone metabolism and ^18^F-PET scan (visit 2); a bone biopsy following a standardized tetracycline labeling period (visit 3); and a final visit for confirmation of wound healing and removal of sutures at the site of biopsy (visit 4). Written informed consent was obtained from all participants, and the study was approved by the local Research Ethics Committee and the UK Administration of Radioactive Substances Advisory Committee.

### Measurements of BMD and Laboratory Assessments

DXA scans were performed at the lumbar spine (L1–L4), left hip including femoral neck and total hip, and nondominant forearm (Hologic Discovery; Hologic, Bedford, MA).

Routine laboratory tests were performed at screening to assess serum calcium, albumin-corrected calcium; alkaline phosphatase; phosphate, renal, and thyroid profiles; full blood count; coagulation screen; PTH; and 25-hydroxyvitamin D. All tests were performed by the local hospital laboratory.

### Biochemical Markers of Bone Turnover

Nonfasting serum samples were collected during early morning for all subjects at visit 2 for the assessment of iPTH, 25-hydroxyvitamin D, bone-specific alkaline phosphatase (BSAP), procollagen propeptide of type 1 collagen (PINP), tartrate-resistant acid phosphatase 5b (TRAP5b), osteoprotegerin (OPG), and fibroblast growth factor-23 (FGF-23). BSAP was measured using the Access^®^ automated immunoassay (Beckman Coulter, Brea, CA): interassay precision was 4.8 %. PINP was measured using the Cobas e411 automated immunoassay (Roche Diagnostics, Penzberg, Germany): interassay precision was 3.8 %. TRAP5b was measured using an ELISA (Immunodiagnostic Systems, Boldon, UK): interassay precision was 7.4 %. Serum OPG was measured using an ELISA (Biovendor, Brno, Czech Republic): interassay precision was 6.9 %. Serum FGF-23 measured using an ELISA (Immutopics, San Clemente, CA): interassay precision was 4.7 %. Samples were stored at −70 °C and analyzed as one batch by a central laboratory at the end of the study.

### Bone Biopsy and Histomorphometry

Transiliac crest bone biopsies were performed following a standardized tetracycline-labeling schedule. The labeling schedule consisted of a 2-day oral administration of demeclocycline hydrochloride (300 mg bid), which started 17 days prior to biopsy, followed by a drug-free interval of 10 days and a further 2-day oral administration of demeclocycline hydrochloride (300 mg bid). Bone biopsy was performed 4 days after completion of the second label. All subjects were provided with labeling instructions at visit 2 and contacted by telephone just prior to each labeling period to ensure compliance with the dosing schedule. A total of 8 of the 12 subjects in the osteoporosis group underwent a successful bone biopsy procedure. Two subjects failed the coagulation screen performed at visit 2 as part of the prebiopsy safety evaluations: one subject had an allergic reaction to demeclocycline following the first dose, and it was not possible to perform the bone biopsy procedure on one subject, following sedation, due to gross adipose tissue overlying the biopsy site.

Transiliac bone biopsies were obtained under local anesthesia and conscious sedation (1 % lignocaine and midazolam, respectively) using an 8-mm internal diameter manual trephine system. Bone biopsies were fixed in 70 % ethanol and subsequently dehydrated in increasing concentrations of ethanol up to 100 %. Biopsies were then embedded, undecalcified, in LR white medium resin (London Resin, London, UK). Sections were cut using a Bright 5040 microtome (Bright Instruments, Huntingdon, UK): 8-μm sections were taken for staining with the von Kossa technique, and 12-μm sections were mounted unstained for fluorescence studies. Composite digital images of whole sections were made in two ways: von Kossa images at ×4 objective were montaged using an Olympus (Tokyo, Japan) BH2 microscope, a Q-Imaging digital camera, a manual stage, and the Bioquant (Nashville, TN) manual imaging toolkit; fluorescent images at ×10 objective using a Leica (Solms, Germany) microscope, a Q-Imaging digital camera, a Prior automated stage, and Surveyor (Objective Imaging, Cambridge, UK) software. Images were analyzed using Bioquant Osteo II software: at least three sections across each biopsy were analyzed (bright field and fluorescent measurements) and mean values estimated. To define the trabecular region of interest (ROI), a rectangular box was drawn for each section which included the majority of the trabeculae but left a clear margin between the box and the endocortical surface at each side left and right and the box and the edge of the section top and bottom. Parameters measured on bright field were tissue volume (TV), osteoid volume (OV), bone volume (BV), osteoid thickness (O.Th), and bone surface (BS). From these measurements, indices were calculated including BV/TV (%), OV/BV (%), and OV/TV (%). Within the ROI on the fluorescent slides single and double labels were traced from which mineralizing surface (MS) was calculated automatically as the double plus half the single tetracycline-labeled surface. Using the bone surface measurement from the corresponding bright field slide, MS/BS (%) was calculated. Where double labels were present the MAR (micrometers per day) was automatically calculated. In biopsies where single but no double labels were detected, a value for MAR of 0.3 μm/day was assigned (model 1) or excluded from the analysis together with biopsies in which no labels were detected (model 2). BFR was calculated from the equation [17]

1 $$ {\text{BFR}}/{\text{BS}} = {\text{MAR}} \times ({\text{MS}}/{\text{BS}}/100)(\mu {\text{m}}^{3} /\mu {\text{m}}^{2} /{\text{day}}).$$

All biopsies were prepared and assessed blinded by one observer.

### ^18^F-Fluoride Positron Emission Tomography

^18^F-PET scans were acquired and analyzed using the methods described by Siddique et al. [39, 40]. Scans were acquired on a GE Discovery PET/CT scanner (General Electric Medical Systems, Waukesha, WI) with a 15.4-cm axial field of view. Following an intravenous injection of 90 MBq ^18^F-fluoride, a 60-min dynamic scan of the lumbar spine was acquired, followed by a 30-min whole-body scan from skull to mid-femur. Low-dose CT scans were performed for attenuation correction and image segmentation. The following bone ROIs were segmented using the CT scan images for the PET scan analysis: lumbar spine (L1–L4), thoracic spine (T1–T12), cervical spine (C1–C7), total hip, femoral neck, femoral shaft, pelvis, humerus, forearm, and calvaria. The total-hip, femoral neck, femoral shaft, humerus, and forearm ROIs were the mean of the two sides. The total-hip and femoral neck ROIs were equivalent to the regions used for DXA hip scan analysis. The femoral shaft ROI was a 60-mm-long annular cylindrical section of cortical bone in the femoral shaft measured from just below the lesser trochanter and excluding the medullary cavity. The forearm was the average of the radius and ulna. The calvaria was defined as the region of the skull above the orbitomeatal line. The regions defined on the CT images were projected onto the PET scans to determine the bone time activity curve (kilobecquerels per milliliter) for the lumbar spine dynamic scan and the average activity concentration (kilobecquerels per milliliter) in each of the other nine ROIs on the whole-body scan.

The arterial plasma input function was estimated using a semipopulation method [41]. Venous blood samples were collected at 30, 40, 50, and 60 min following injection to define the terminal exponential for each individual. For each subject in the present study, the population residual curve based on direct arterial sampling in ten postmenopausal women was scaled for injected activity and added to the terminal exponential curve to obtain the arterial plasma input function used for kinetic analysis [35, 41].

The dynamic PET scan and blood data for each subject were used to estimate the bone plasma clearance (*K*_i_) at the lumbar spine by applying Patlak analysis to the 10- to 60-min data points [39]. At other skeletal sites values of *K*_i_ were estimated using the single-point Patlak method of Siddique et al. [40] assuming Patlak plot intercepts of 0.44 and 0.10 for the spine and nonspine regions, respectively. A correction was made for tracer efflux from bone from the time of injection to the midpoint of the relevant bed position using the method described by Siddique et al. [40]. To account for the partial volume effect, a rod phantom experiment was performed to estimate recovery coefficients for each skeletal ROI. Published data on typical bone sizes supplemented by measurements made from DXA scans were used to estimate average bone size at each ROI. Based on this experiment, the recovery coefficients ranged from 0.53 at the calvaria to 0.99 at the total hip and lumbar spine. For all the spine and hip ROIs the recovery coefficients were >0.9 [42].

Since there was a wide variation in the DXA BMD values both between and within the two study groups, the *K*_i_ measurements at the lumbar spine were normalized to site-matched regional bone mass using the DXA measurement of lumbar spine BMD corrected for vertebral body size, bone mineral apparent density (BMAD) [43], thus providing a measure of the plasma clearance of ^18^F-fluoride per gram of bone tissue, calculated as

2 $$ K_{{{\text{i}}/{\text{BMAD}}}} = K_{\text{i}} /{\text{BMAD}}\,{\text{mL}}/\hbox{min} /{\text{g}}.$$

### Statistical Analysis

The bone histomorphometric, bone turnover marker (BTM), and ^18^F-PET parameters were all tested for normality using the Shapiro–Wilk test. Many of the parameters failed the test for normality, and therefore, nonparametric statistical tests were applied. Values for BMD, *K*_i_, and *K*_i/BMAD_ were expressed as mean and standard deviation (SD). Values for bone histomorphometric parameters and BTMs were expressed as median and interquartile range (IQR). Differences between the CKD5D and osteoporosis groups were evaluated using a Mann–Whitney *U*-test. A Friedman’s two-way analysis of variance was used to examine differences in *K*_i_ between skeletal sites. To allow for multiple comparisons the statistical significance of the differences between pairs of sites (limited to comparison of the key sites of lumbar spine, total hip, and forearm) was evaluated using the Wilcoxon signed-rank test with a Bonferroni correction. Correlations between ^18^F-PET, bone histomorphometric parameters, and BTMs were assessed using the Spearman rank correlation test. *p* ≤ 0.05 was considered statistically significant.

## Results

Study group characteristics are shown in Table 1. The mean age of subjects in the CKD5D and osteoporosis groups was 64 and 65 years, respectively. Six of the seven subjects in the CKD5D group were male, all were on hemodialysis, and the average duration of dialysis was 11.4 years. Three of the seven CKD5D subjects had a history of parathyroidectomy (4, 13, and 29 years prior to study participation), six were on active vitamin D therapy in the form of alpha-calcidol, and three were receiving phosphate binders. All seven of the CKD5D patients had iPTH levels <150 pg/mL as per protocol, and six of these subjects had levels <100 pg/mL. Serum calcium levels were significantly lower in the CKD5D group compared to those in the osteoporosis group. Levels of PINP, OPG, and FGF-23 were significantly higher in the CKD5D group. The mean lumbar spine BMD *T* score was −1.0 and −2.4 for the CKD5D and osteoporosis groups, respectively.

**Table 1:** Study group characteristics

Variable	CKD5D	Osteoporosis	Reference range
*n*	7	12	
Age (years)	64.0 (15.4)	65.0 (7.4)	
Male/female (*n*)	6/1	0/12	
Previous fracture (*n*)	2/7	7/12	
eGFR (mL/min/1.73 m^2^)	11.1 (7.3)***	86.4 (10.8)	
Duration of dialysis (years)	11.4 (11.4)	–	
Vitamin D therapy (*n*)	6/7	–	
Phosphate binders (*n*)	3/7	–	
Serum iPTH (pg/mL)	45.2 (40.0)	43.4 (16.4)	
Serum 25-hydroxyvitamin D (ng/mL)	19.9 (3.9)	22.6 (6.8)	
Serum calcium (mmol/L)	2.21 (0.20)*	2.39 (0.11)	
Serum phosphate (mmol/L)	1.09 (0.33)	1.24 (0.14)	
Serum alkaline phosphatase (IU/L)	130.4 (73.7)	80.42 (30.7)	
Serum BSAP (μg/L)	18.9 (15.1)	15.1 (8.3)	5.15–15.32 [58]
Serum PINP (ng/mL)	192.5 (592.2)***	62.9 (26.6)	16.3–78.2 [58]
Serum TRAP5b (U/L)	2.3 (3)	3.3 (2.5)	1.2–4.4 [59]
Serum OPG (pmol/L)	17.4 (23.0)***	5.5 (2.3)	
Serum FGF-23 (RU/mL)	695.2 (1333.1)***	69.3 (37.4)	
Lumbar spine BMD *T* score	–1.04 (2.40)	–2.40 (1.44)	
Total-hip BMD *T* score	–1.09 (1.15)	–1.80 (0.51)	
Total forearm BMD *T* score	–2.23 (2.35)	–2.61 (1.44)	

Data are expressed as mean and SD except the biochemical marker data, which are expressed as median and IQR

*eGFR* estimated glomerular filtration rate, *iPTH* intact parathyroid hormone, *BSAP* bone-specific alkaline phosphatase, *PINP* procollagen propeptide of type 1 collagen, *TRAP* tartrate-resistant acid phosphatase 5b, *OPG* osteoprotegerin, *FGF-23* fibroblast growth factor-23, *BMD* bone mineral density

* *p* < 0.05, ** *p* < 0.01, *** *p* < 0.001 versus osteoporosis group calculated using Mann–Whitney *U*-test

### Bone Histomorphometry

Bone histomorphometric results are shown in Table 2. Of the seven biopsies collected from the CKD5D patients only two had evidence of double tetracycline labels, with the remaining five samples having single labels only. A default value of 0.3 μg/day was assigned for the MAR if only single labels were present (model 1 described in “Methods” section). All biopsy samples in the osteoporosis group had double labels, with the exception of one sample which was devoid of labels. Median values were significantly lower in the CKD5D group compared to those with osteoporosis for both MAR (0.30 vs. 0.61 μm/day, *p* < 0.001) and BFR/BS (0.002 vs. 0.010 μm^3^/mm^2^/day, *p* < 0.001). Median values for MS/BS % were also significantly lower in the CKD5D group (0.55 vs. 2.20 %, *p* < 0.05). Qualitative evaluation of the seven bone biopsy samples from the CKD5D patients classified them into three subtypes of bone disease: ABD (*n* = 2), mixed uremic osteodystrophy (*n* = 4), and severe osteomalacia (*n* = 1).

**Table 2:** Bone histomorphometric parameters for individual CKD5 patients

	Tetracycline label	MAR (μm/day)^a^	BFR/BS (μm^3^/mm^2^/day)	OS/BS (%)	MS/BS (%)	Clinical interpretation
CKD5D patients						
1	Single	0.30	0.002	13.40	0.55	Mixed
2	Single	0.30	0.001	0.65	0.30	Adynamic bone disease
3	Single	0.30	0.010	62.40	3.40	Severe osteomalacia
4	Double	0.28	0.005	8.60	0.78	Mixed
5	Single	0.30	0.002	1.17	0.67	Adynamic bone disease
6	Double	0.49	0.001	61.20	0.53	Mixed
7	Single	0.30	0.001	8.60	0.38	Mixed
Median (IQR)	–	0.30 (0.00)***	0.002 (0.004)**	8.60 (60.03)	0.55 (0.40)*	
Osteoporosis						
Median (IQR)	6 Double/1 absent labels	0.61 (0.21)	0.010 (0.030)	7.25 (4.96)	2.20 (5.80)	–

^a^For biopsies with single tetracycline labels only, a default value of 0.3 μg/day was assigned for the MAR (model 1 described in “Methods” section) [17]

*Mixed* combination of increased bone turnover and a mineralization defect

* *p* < 0.05, ** *p* < 0.01, *** *p* < 0.001 versus osteoporosis group calculated using the Mann–Whitney *U*-test

Subjects 2 and 5 in Table 2 had ABD. Subject 2 was an 80-year-old white male with a history of chronic hypertensive renal disease; he had received hemodialysis for 1.2 years and had an iPTH measurement of 79 pg/mL and the lowest values of BSAP and PINP compared to the other CKD5D patients. Subject 5 was a 48-year-old black male with pyelonephritis; he had received dialysis for 20 years and had the lowest iPTH value of 6 pg/mL, a high PINP level of 630 ng/mL, BSAP values above the normal reference range, and TRAP5b values within the normal reference range.

### Qualitative and Quantitative Measurements of Bone Formation Using ^18^F-PET

**Fig. 1:**
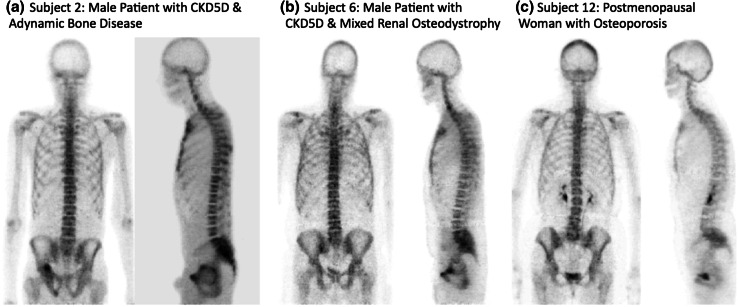
^18^F-PET scans showing **a** coronal and sagittal views for subject 5, a 48-year-old black male with CKD5D and adynamic bone disease; **b** coronal and sagittal views for subject 6, a 46-year-old white male with CKD5D and mixed uremic osteodystrophy; **c** coronal and sagittal views for subject 12, a 68-year-old white postmenopausal woman with osteoporosis

**Fig. 2:**
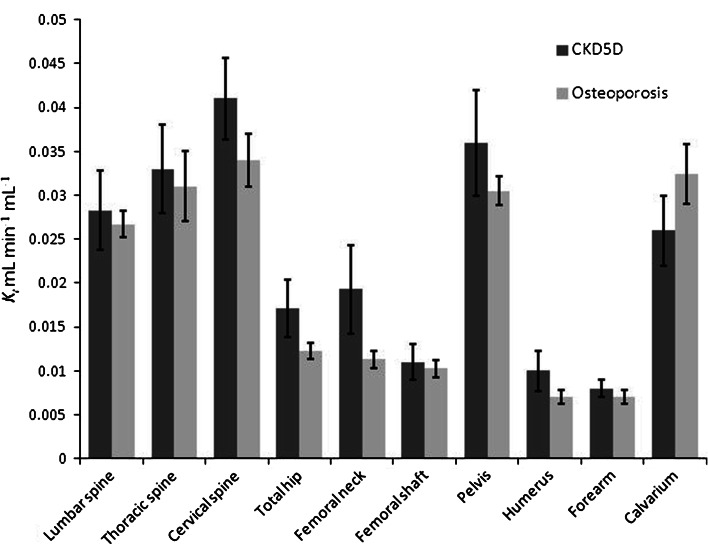
^18^F-PET measurements of *K*_i_ at multiple skeletal sites. Significant differences in *K*_i_ between different skeletal sites were observed for both the CKD5D (*p* < 0.001) and osteoporosis (*p* < 0.001) groups. No significant differences were observed between the CKD5D and osteoporosis groups for mean *K*_i_ at each skeletal site

Qualitative assessment of the individual ^18^F-fluoride whole-body scans for all subjects did not show any discernible differences in image quality or skeletal uptake of ^18^F-fluoride between those in the CKD5D and osteoporosis groups (Fig. 1), including the two CKD5D subjects with ABD. Mean ^18^F-PET measurements of bone formation (the plasma clearance of ^18^F-fluoride to bone, *K*_i_) at all measured skeletal sites for each of the study groups are shown in Fig. 2. Individual and mean values of *K*_i_ for the CKD5D group at the main skeletal sites are compared with mean values for those with osteoporosis in Table 3. There was significant heterogeneity in bone formation between skeletal sites for both study groups (Table 3; Fig. 2) that was confirmed statistically (Friedman’s two-way analysis of variance *p* < 0.001 for both the CKD5D and osteoporosis groups). The pattern of skeletal heterogeneity was similar between the two groups (Fig. 2). At each skeletal ROI no significant difference in *K*_i_ between the CKD5D and osteoporosis groups was found. The variability (and range) of individual *K*_i_ results tended to be greater in the CKD5D group, as demonstrated by the higher SD values compared to those obtained for the osteoporosis group. Focusing on CKD5D subjects 2 and 5 with confirmed ABD, subject 2 had the lowest values of *K*_i_ at the lumbar spine, total hip, and pelvis and the second lowest results at the humerus, forearm, and BMAD-corrected *K*_i_ values at the lumbar spine; subject 5 had the highest values of *K*_i_ at the lumbar spine and the second highest at nonspine sites (Table 3).

**Table 3:** ^18^F-PET measurements of regional bone formation at key skeletal sites

	*K*_i_ (mL/min/mL)						*K*_i/BMAD_ (mL/min/g)
	Lumbar spine	Total hip	Femoral shaft	Pelvis	Humerus	Forearm	Lumbar spine/BMAD^a^
CKD5D patients							
1	0.034	0.016	0.009	0.033	0.009	0.010	0.166
2^b^	0.021	0.009	0.009	0.020	0.007	0.006	0.088
3	0.022	0.009	0.005	0.026	0.006	0.005	0.108
4	0.028	0.035	0.020	0.060	0.019	0.011	0.079
5^b^	0.053	0.020	0.019	0.053	0.015	0.010	0.163
6^c^	0.022	0.014	0.009	0.034			0.083
7	0.018	0.018	0.009	0.028	0.007	0.007	0.084
Mean (SD)	0.028 (0.012)	0.017 (0.009)	0.011 (0.006)	0.036 (0.015)	0.010 (0.006)	0.008 (0.003)	0.111 (0.038)
Osteoporosis							
Mean (SD)	0.027 (0.005)	0.012 (0.003)	0.010 (0.003)	0.031 (0.006)	0.007 (0.003)	0.007 (0.003)	0.121 (0.018)

^a^ *K*_i_ normalized to site-matched regional bone mass using bone mineral apparent density (see “Methods” section for full description)

^b^Adynamic bone disease confirmed on biopsy

^c^Measurement of *K*_i_ not obtained at humerus or forearm as both arms outside of field of view during scan acquisition

*K*_i_ = the plasma clearance of ^18^F-fluoride to bone mineral (see “Methods” section for full description)

### Correlation Between ^18^F-PET, Bone Histomorphometry, and Biochemical Markers

**Fig. 3:**
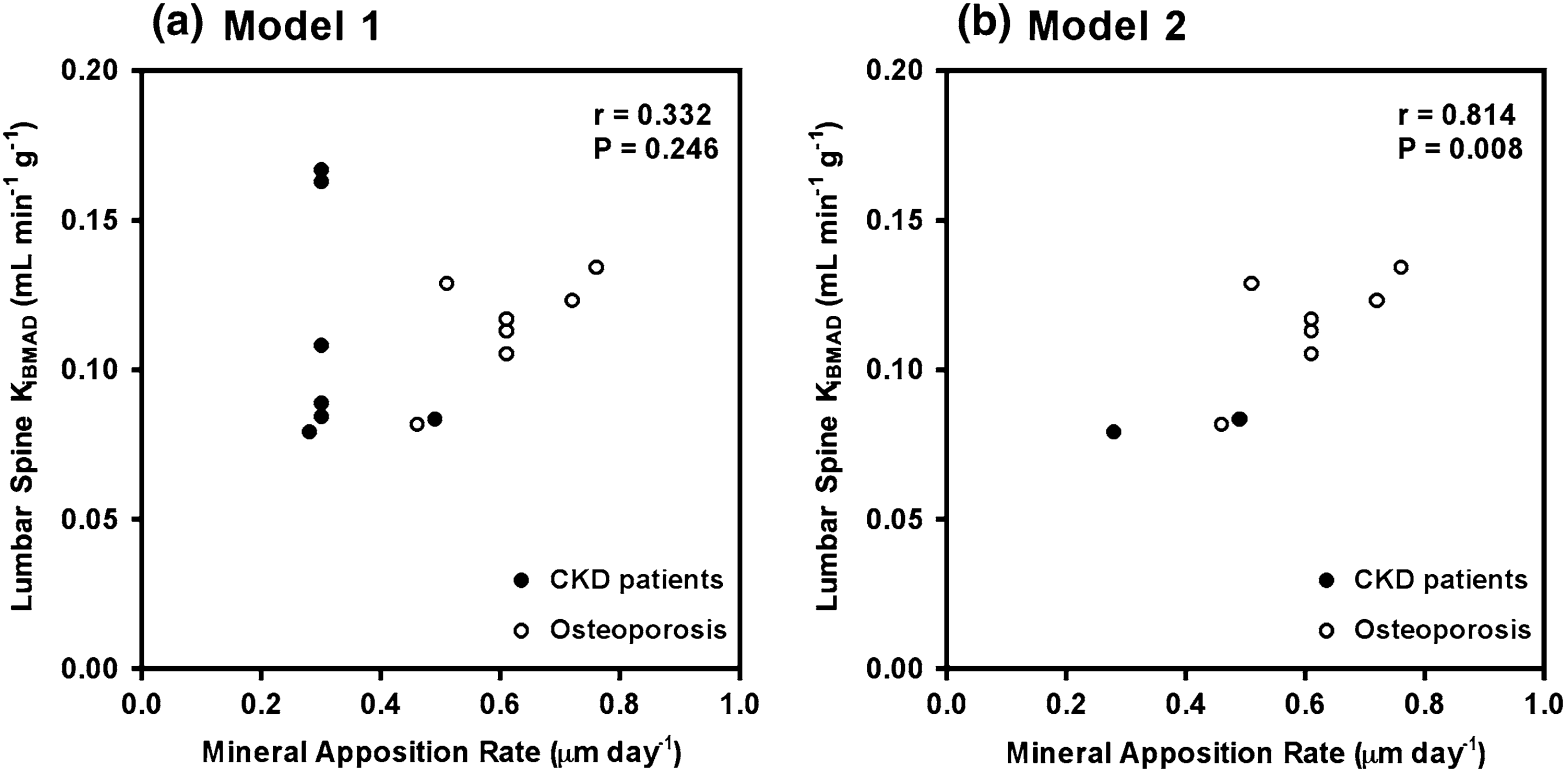
*Scatter plots* showing the relationship between *K*_i/BMAD_ and mineral acquisition apposition rate using **a** model 1, including biopsies with single and double tetracycline labels, and **b** model 2, excluding biopsies with single or no tetracycline labels

^18^F-PET measurements of *K*_i_, measured at the lumbar spine and corrected for BMAD, were directly compared to the four bone histomorphometric parameters, and the results are shown in Table 4, using a default value of 0.3 μm/day for MAR for biopsies with single labels only (model 1) and excluding biopsies with single labels (model 2), described in full in “Methods” section. Using model 1, there were no significant correlations between *K*_i/BMAD_ and any of the four bone histomorphometric parameters. For model 2, a significant correlation between *K*_i/BMAD_ and MAR was observed (*r* = 0.81, *p* = 0.008) but not between *K*_i/BMAD_ and BFR/BS (*r* = 0.59, *p* = 0.092) (Table 4). Figure 3 shows the scatter plots of *K*_i/BMAD_ against MAR. Two of the seven CKD patients had high values for *K*_i/BMAD_ but a low value for MAR of 0.30 μm/day as only a single tetracycline label was present, i.e., model 1 (Fig. 3a). These two outliers have an adverse impact on the correlation between *K*_i/BMAD_ and MAR as shown by the high correlation (*r* = 0.81, *p* = 0.008) between these two parameters when subjects with single labels (including the two outliers) are excluded using model 2 (Fig. 3b). There were no significant correlations between *K*_i_, uncorrected for BMAD, at any skeletal site and either BFR or MAR.

**Table 4:** Correlation between bone histomorphometric parameters and ^18^F-PET at the lumbar spine

*K*_i/BMAD_ lumbar spine^a^	*n*		MAR (μm/day)	BFR/BS (μm^3^/mm^2^/day)	MS/BS (%)	OS/BS (%)
Model 1	14	*r*	0.33	0.30	0.23	–0.05
		*p*	0.246	0.298	0.427	0.864
Model 2	9	*r*	0.81	0.59	0.55	–0.09
		*p*	0.008	0.092	0.125	0.803

^a^ *K*_i_ measured at the lumbar spine corrected for volumetric BMD (BMAD) estimated using site-matched areal DXA scans

Model 1 included biopsies with double labels and single labels. A default value of 0.30 μm/day was assigned to biopsies with single labels only. Model 2 excluded biopsies with single or no labels. Data represent the correlation coefficient (*r*) and *p* values calculated using the Spearman rank test

When the bone histomorphometric and BTM results were compared, there was a significant correlation between BFR/BS and PINP (*r* = 0.95, *p* = 0.001), OPG (*r* = 0.77, *p* = 0.044), and TRAP5b (*r* = 0.77, *p* = 0.044) for the CKD5D group, while MAR correlated significantly with OPG (*r* = 0.78, *p* = 0.039) for the osteoporosis group. No significant correlations were observed between *K*_i_ at any site and BTM results for either the CKD5D or osteoporosis group.

## Discussion

As few nephrologists have access to specialized histopathological services [44], bone biopsy remains unrealistic in many centers; and this, combined with the known relatively poor predictive capabilities of a single measurement of serum iPTH [20], provided the rationale for this study. The current study was limited by the small sample size, but it has demonstrated that ^18^F-PET can be used to assess regional bone formation at multiple sites of the skeleton in CKD5D patients, including clinically relevant sites such as the spine, hip, and forearm, and allows a comparison of cortical and trabecular bone. In addition, a significant correlation was observed between *K*_i_, corrected for BMAD, and MAR derived using the gold standard of bone biopsy at the iliac crest.

The CKD5D patients were identified as having suspected ABD if serum iPTH was consistently below the threshold of <150 nmol/L in the preceding 3 months prior to screening. Subsequent evaluation of the bone biopsy samples revealed that only two subjects (numbers 2 and 5 in Table 2) had ABD confirmed by bone histology as evident by low numbers of bone cells, low bone formation, and reduced osteoid amount. Significant correlations, typically in the range 0.5–0.7, between iPTH measurements and bone histology have been reported [45–48]. However, it is accepted that the utility of iPTH for correctly classifying bone subtypes in individual subjects is limited [19, 20]. Therefore, the fact that only two of the CKD5D patients had confirmed ABD on bone histology is not entirely unexpected. Furthermore, the diagnosis of ABD is complex, even when using bone histomorphometry. A recently published report demonstrated this in two hemodialysis patients with very low PTH levels and suspected ABD [49]. Histomorphometric parameters in cancellous bone confirmed ABD in both patients using conventional classification. However, bone remodeling was increased at all three surfaces in cortical bone, evident by increased osteoblastic surface, osteoid surface, BFR, and other parameters [49]. It has been suggested that many cases of ABD based on cancellous bone, with the exclusion of cortical bone, should be more appropriately considered patients with low to normal turnover [50, 51]. It is also clear that ABD should not be considered one entity with a uniform histological picture. There may be a complete lack of bone cells and no tetracycline labels in some samples but evidence of bone cells and single or double labels indicating bone-remodeling activity in others. In addition, bone status does not remain static during the course of CKD, transitioning from high to low turnover, and vice versa, due to underlying changes in mineral derangements or in response to treatment [52].

No qualitative differences were noted between ^18^F-PET scans acquired on patients in the CKD5D group and those in the osteoporosis group (Fig. 1). This is important since the tracer ^18^F-fluoride is preferentially deposited at sites of osteoblastic activity and mineralization and, theoretically at least, image quality may have been impaired due to low tissue to background ratio for conditions where bone formation and/or mineralization are severely diminished. More interestingly, it was not possible to qualitatively distinguish the two patients with confirmed ABD from those with mixed renal osteodystrophy or those with osteoporosis (Fig. 1). The fact that there was skeletal uptake of ^18^F-fluoride in all patients with CKD5D confirms that bone formation and subsequent mineralization were occurring; i.e., BFR and MAR were not zero, albeit presumably at a diminished rate in those with ABD. This is confirmed by the presence of at least one tetracycline label in all CKD5D subjects (Table 2). As emphasized in a review by Recker et al. [18], even individuals with BFRs of zero at the iliac crest measured using histomorphometry have normal to low biochemical markers of bone formation that never, or very rarely, reach zero. Evidence using ^18^F-PET imaging is very limited, but other studies of patients with low bone turnover as a consequence of renal bone disease [26] or glucocorticoid use [36] have not reported issues with poor image quality.

The lack of a qualitative difference in ^18^F-PET scans between those with CKD5D and osteoporosis is confirmed quantitatively, with no significant differences in mean *K*_i_ between the CKD5D and osteoporosis groups at any of the skeletal sites measured (Table 3). However, one should express caution about directly comparing these two groups. The histological features of ABD are similar to other disorders associated with low formation rates, including some cases of postmenopausal osteoporosis [53]. The mean *K*_i_ results obtained at the lumbar spine and hip ROIs for the osteoporosis group were similar to those previously reported for treatment-naive postmenopausal women with osteopenia and osteoporosis [35, 36]. Values of *K*_i_ obtained at the lumbar spine for the CKD5D group were similar to those obtained in a study of CKD5D patients with low bone turnover (Table 3) [26]. As described in “Results section,” the ^18^F-PET results for the two subjects with ABD were conflicting. The ^18^F-PET and biochemical marker results for subject 2 are consistent with his confirmed diagnosis of ABD. In contrast, subject 5 had *K*_i_ and PINP values suggestive of increased bone turnover, which is at odds with his diagnosis of ABD (Table 2). Whether these discordant results demonstrate a potential lack of diagnostic accuracy of ^18^F-PET, a limitation associated with performing biopsies at only one skeletal site of trabecular bone only, or a combination of both cannot be established. A larger study of patients with CKD is therefore warranted to determine the value of ^18^F-PET as a diagnostic tool. Patients with low iPTH levels suggestive of ABD and who do not undergo a biopsy for whatever reason almost always remain untreated due to the uncertainty regarding the safety of an antiresorptive treatment. The quantitative assessment of bone formation at clinically relevant sites using ^18^F-PET could prove most useful in ruling out ABD so that a decision regarding treatment can be made.

One important issue highlighted in this study is how to deal with bone biopsy samples that include single labels only. Five of the seven CKD5D subjects had only a single tetracycline label evident in the bone biopsy sample (Table 2). Although it cannot be completely ruled out, since one label was seen in these patients rather than no labels, it is unlikely that this was due to patients not complying with the labeling schedule. Single labels are common when remodeling rates are low, such as in patients with CKD and evidence of ABD [54] and patients with osteoporosis treated with potent antiresorptive therapy [55–57]. In a biopsy substudy of the phase III trial of denosumab, double labeling in trabecular bone was observed in only 19 % of samples and no labels were observed in 66 % of samples [57]. Of note, in the context of the present study, was the similar biochemical marker results obtained in those with absent versus double tetracycline labels in the denosumab trial [57], highlighting the complexities of interpreting bone biopsies obtained from one very small site of the skeleton and making comparisons with global markers of bone remodeling. In line with the recent ASBMR Histomorphometry Nomenclature Committee recommendations, for biopsy samples with only single labels a minimum value for MAR of 0.3 μm/day was imputed (Table 2). For subsequent statistical analysis of the correlation between ^18^F-PET and bone histomorphometry, biopsies with single labels assigned a minimum value for MAR were either included (model 1) or not included, i.e., considered missing values (model 2) [17].

A significant correlation between plasma clearance of ^18^F-fluoride at the lumbar spine corrected for BMAD (*K*_i/BMAD_) and MAR was observed in the present study but only when using model 2 (*r* = 0.81, *p* = 0.008; Table 4, Fig. 3). Two of the CKD patients, one with ABD (subject 5) and one with mixed uremic osteodystrophy (subject 1), had high values for *K*_i/BMAD_ at the lumbar spine but a low value of 0.3 for MAR as both had single tetracycline labels only (Fig. 3a). These outliers had an adverse impact on the correlations between ^18^F-PET and bone biopsy (Table 4). This further highlights the problems of performing bone biopsies in low-turnover settings and the difficulties that may arise when comparing measurements taken at different skeletal sites and using different methodologies. Nevertheless, the significant correlation between *K*_i/BMAD_ and MAR is consistent with those obtained in a previous clinical study of dialysis patients with CKD5 [26] and an animal study [27], with the former reporting a correlation coefficient of 0.84 between *K*_i_ at the thoracic spine and BFR [26] and the latter reporting a correlation coefficient of 0.81 between *K*_i_ at the lumbar spine and MAR [27]. These studies [26, 27] and the current study were all small in size. Further, in the current study no significant correlations were observed between *K*_i_, uncorrected for BMAD, and any of the histomorphometric parameters. Since the clearance of any bone-seeking tracer could theoretically be dependent, at least in part, on the surface area of hydroxyapatite available for tracer exchange, an attempt was made to correct for this using BMAD as a surrogate measure of bone surface area. This was particularly important in the current study since the variability in BMD values between individuals was very high (e.g., lumbar spine *T* score range −4.5 to +2.4). Whether correcting plasma clearance measurements for volumetric BMD measured by CT or BMAD using DXA as surrogates of bone surface area enhances diagnostic accuracy or sensitivity for assessing treatment efficacy has not yet been tested. However, the correlation between *K*_i/BMAD_ at the lumbar spine and MAR (Table 4) is encouraging and warrants further investigation in a larger study.

This study has a number of limitations. The sample size was very small owing to the difficulty in recruiting patients in a study which involved having a bone biopsy, and this was further confounded by the lack of double tetracycline labels in biopsies from five of the seven CKD5D patients. The absence of MAR data for these five biopsies meant that the statistical analysis required the use of two alternative conventions for handling missing data, model 1 or model 2, either imputing values for the missing results or excluding these subjects from the data analysis. The correlations reported in Table 4 are dependent on these two conventions, and it is unclear whether, were the true MAR results measurable with larger biopsy samples, what effect this would have on these results. Although no correlations were found between the raw *K*_i_ results and bone histomorphometry, a relationship was found when lumbar spine *K*_i_ was corrected for site-matched estimated volumetric BMD (BMAD). While this correction can be justified because of the differences in lumbar spine *T* score between the CKD5D and osteoporosis groups (Table 1), there is presently no published evidence to support the correction of *K*_i_ values using BMAD.

In the management of CKD a bone biopsy may be essential for certain individuals or in particular clinical circumstances, and even with its well-documented limitations, it remains the gold standard for diagnosis [14]. This study highlights the potential of ^18^F-PET as a noninvasive imaging tool for the assessment of regional bone formation in patients with bone abnormalities associated with CKD. The significant correlation between ^18^F-PET and MAR was encouraging. However, the discordant results between iPTH, biochemical markers, and ^18^F-PET and underlying bone histology are difficult to interpret in this small study. Further studies are required to establish the sensitivity of ^18^F-PET as a diagnostic tool for identifying those with ABD. Such a study would also need to demonstrate that sensitivity of ^18^F-PET is superior to that of circulating iPTH concentrations to justify its extra cost and complexity.
